# Improving legislation is a necessity: A multi-method quantitative analysis of tobacco sponsorship and publicity in China

**DOI:** 10.18332/tid/217354

**Published:** 2026-03-24

**Authors:** Shuyue Sun, Yimeng Wu, Yixin Tang, Fan Wang

**Affiliations:** 1 School of Public Health, Fudan University, Shanghai, China; 2 School of Public Policy and Management, Tsinghua University, Beijing, China; 3 College of Foreign Languages and Literature, Fudan University, Shanghai, China; 4 School of Journalism, Fudan University, Shanghai, China; 5 Fudan Development Institute, Fudan University, Shanghai, China; 6 Health Communication Institute, Fudan University, Shanghai, China

**Keywords:** tobacco sponsorship, China, law, media, publicity, corporate social responsibility

## Abstract

**INTRODUCTION:**

Although the WHO Framework Convention on Tobacco Control (FCTC) mandates a comprehensive ban, tobacco sponsorship persists as a significant challenge in China. Focusing on this issue, this study aims to describe, identify, and understand the amounts, publicity, and influencing mechanism of tobacco sponsorship in China.

**METHODS:**

This multi-method quantitative study collected data from the China Tobacco Yearbooks for provincial tobacco sponsorship and production data, the Chinalawinfo Open Platform for anti-smoking regulations, and four tobacco industry websites for publicity materials, covering 2015 to 2022. Using ArcMap, STATA, and Python, we integrated spatial autocorrelation analysis, topic modeling, content analysis, and statistical modeling.

**RESULTS:**

Tobacco sponsorship amounts nationwide plummeted in 2017, followed by a gradual recovery until another decline in 2021. Spatial analysis revealed significant positive spatial autocorrelation from 2016–2020 and in 2022 (Global Moran’s I>0.1, p<0.05). Sponsorship publicity predominantly centered on four topics: natural and health disaster response, tobacco product production and manufacturing, poverty alleviation and policy response, and customer service and brand building. OLS regression indicated that smoke-free law efficacy was negatively associated with sponsorship amounts (β= -0.149; 95% CI: -0.238 – -0.060; p<0.01), while cigarette production showed a positive association (β=0.001; 95% CI: 0.001–0.002; p<0.01). These associations remained robust after controlled for government-industry connection and production chain linkage. Mediation analysis further suggested that ‘Mentioning tobacco farmers’ served as a significant negative mediator for the impact of SLE (indirect effect= -0.351; 95% CI: -0.603 – -0.099; p<0.01) and a significant positive mediator for cigarette production (indirect effect=0.001; 95% CI: 0.000–0.001; p<0.05), government-industry connection (indirect effect=0.138; 95% CI: 0.084–0.192; p<0.01), and production chain linkage (indirect effect=0.338; 95% CI: 0.255–0.420; p<0.01). Robustness checks using heteroscedasticity-consistent and province-clustered standard errors confirmed the stability of these findings.

**CONCLUSIONS:**

Tobacco sponsorship is fundamentally profit-driven because it mainly funds and publicizes components of the production chain, particularly tobacco farmers. As its severity may be constrained by anti-tobacco legislation, future studies are needed to continuously monitor these evolving strategies, thereby accumulating sufficient evidence to support the introduction and implementation of comprehensive, nationwide legislation that clearly defines and penalizes all sponsorship activities in China.

## INTRODUCTION

Tobacco sponsorship persists as a significant global challenge hampering tobacco control that the money annually allocated to tobacco advertising, promotion, and sponsorship (TAPS) amounts to tens of billions of USD^1^. As a dual-purpose marketing strategy, sponsorship aims to enhance both tobacco product sales and public relations. On the one hand, exposure to tobacco-sponsored events has been shown to correlate with increased smoking initiation and consumption, particularly among adolescents^2^. While on the other hand, tobacco companies use sponsorship to masquerade themselves as socially responsible entities, normalizing their presence while obstructing regulatory reform^3^. The two functions reinforce each other, collectively hindering tobacco control efforts by changing public awareness and behavior simultaneously.

Article 13 of the World Health Organization (WHO) Framework Convention on Tobacco Control (FCTC) requires Parties to introduce a comprehensive ban on TAPS since its introduction in 2003^4^. Despite its inclusion in TAPS, tobacco sponsorship, compared to tobacco advertising and promotion, is still often overlooked. Given the prevailing industrial orientation to concentrate on the upstream (tobacco production) and downstream (cigarette sales) sectors^5^, it is not surprising that sponsorship has been seen as a secondary issue, rather than an independent concern, which makes it remain underexplored. This lack of attention has also been reflected in the regulatory inertia of governments, as targeted restrictions had been hardly developed, largely lagging behind the other two counterparts: While 163 countries have implemented bans on tobacco advertising on national television and radio, and 130 have prohibited promotional discounts, only 73 countries prohibit financial or in-kind tobacco sponsorship, and a mere 6 countries enforce restrictions on Corporate Social Responsibility (CSR)^6^.

As the world’s largest tobacco producer and consumer, China accounts for almost a third of global cigarette consumption, with domestic production exceeding 24.4 trillion cigarettes in 2023 and generating 8.4% of national tax revenue^7-9^. China has shown a high-level compliance with the general TAPS ban; however, its regulations on tobacco sponsorship need to be tightened further, and key measures like a complete ban on CSR activities are still absent^10^. Although the FCTC was ratified in 2005, and the Charity Law was enacted in 2016 and amended in 2023 to restrict tobacco-related philanthropic activities, there is still a lack of a clear definition of tobacco sponsorship and penalties for law violations in China^11^. These loopholes allow tobacco companies to sponsor schools, disaster relief, and cultural programs under the disguise of CSR, effectively circumventing partial advertising bans to embed pro-tobacco messaging in communities^12^.

This regulatory vacuum stems from structural conflicts. On the one hand, the State Tobacco Monopoly Administration (STMA), as ‘the central force in developing and managing China’s tobacco market’^13^, aims to strengthen industry supply chain links to promote brand visibility and sales^14,15^. While on the other hand, though titled ‘the chief manager of anti-tobacco efforts’^13^, it obstructs the progress of anti-tobacco legislation^15^ through its institutional connections with government apparatuses^16^. When the two functions collide, public health welfare in the Chinese tobacco industry tends to be subordinated to commercial interests, impeding the implementation of FCTC measures.

Previous research has primarily focused on tobacco sponsorship activities themselves, neglecting the fact that tobacco industry communications campaigns, a key source of pro-tobacco messaging, are also emphasized in the FCTC. Both the financial and promotional strategies of tobacco sponsorship by Chinese companies remain under-regulated10. Moreover, similar to global assessments of tobacco control laws, there is also a lack of detailed analysis tracking the implementation and enforcement of TAPS bans in China^17^. Notably, the impact of the 2016 Charity Law, which restricts sponsorship publicity, has yet to be studied^11^. Moreover, notwithstanding the 2023 amendment to the Charity Law, previous lack of specific definitions and punitive measures for regulatory violations persists, underscoring the critical necessity for targeted legislative intervention.

By analyzing tobacco sponsorship and publicity through industry yearbooks and corporate websites, our objective was to understand the status quo of sponsorship by Chinese tobacco companies, identify the influencing factors, and contribute empirical insights to support the enforcement of a comprehensive national ban on tobacco sponsorship in China, thereby advancing FCTC implementation in China and globally.

## METHODS

This study utilized a multi-source dataset comprising both statistical records and corporate publicity texts to systematically examine the magnitude and strategies of tobacco sponsorship. While numerical data were utilized to objectively reveal the prevalence and severity of the problem, detailed textual analysis provided a deconstruction of the thematic focus and framing discourse strategies to elucidate the legitimization efforts of the industry. This integrated approach complementarily allows for a comprehensive triangulation of evidence regarding the tobacco sponsorship in China, thereby attempts to identify the statistical determinants of sponsorship amounts and their underlying influencing mechanisms.

### Data collection

*Operational definition of tobacco sponsorship*

Conceptually, in academic research and policy practices, tobacco sponsorship is a broad and inclusive term that encompasses multiple hyponyms referring to diverse forms of corporate support such as business donations, corporate social responsibility (CSR) activities, and other financial or material contributions aimed at public events, individuals, or causes^18^.

Therefore, to ensure coding consistency and reproducibility, we adopted an operational definition of ‘tobacco sponsorship’ grounded in FCTC, which offers a comprehensive range of all TAPS forms. Interpreting from this official definition, we further integrated areas of sponsorship activities and narrative frameworks identified from prior research to refine the inclusion criteria. Specifically, texts were included if their contributions aligned with national policies, public health, CSR, educational support, and economic development, targeting typical areas such as poverty alleviation, pandemic relief, and scholarships and grants.

Moreover, given the complexity and multifaceted characteristics of sponsorship activities, brand visibility served as the paramount criterion for data selection. Any publicity texts that did not directly display tobacco brand names or corporate logos, either within the textual descriptions or supplementary imagery, were excluded from the scope of this study, thereby ensuring clarity and representativeness of the collected samples.

### China tobacco yearbooks

Data from provincial administrative units on tobacco sponsorship and tobacco production were extracted from the China Tobacco Yearbook from 2015 to 2022 (As of March 2025, the latest data available correspond to 2022) – the systematically documenting sector-wide activities by the State Tobacco Monopoly Administration (STMA).

The final dataset constitutes a balanced panel of 8 years and 248 observations; there were no missing values for the core variables (cigarette production and tobacco sponsorship amount).

### Chinalawinfo open platform

We retrieved annual data from the Chinalawinfo Open Platform^19^, a widely used database of Chinese policy documents. Specifically, we tracked the number of active anti-smoking regulations in public spaces (at both provincial and municipal levels) enforced across each calendar year from 2015 to 2022 to assess the evolving national legal landscape.

### Tobacco websites

As the internet has expanded, websites have become critical platforms for tobacco companies to promote themselves. For this study, we searched by keywords of public welfare, charity, donation, sponsorship, funding, infrastructure, and consolation goods, and collected relevant reports from four major tobacco industry publicity websites: Tobacco Online^20^, Xinhua Tobacco Information Network^21^, China Tobacco Media Network^22^, and Oriental Tobacco Network^23^. Of 3063 initially identified articles, 2429 were retained after de-duplication using Python and relevance screening. The screening process was conducted by two researchers working independently to ensure data quality, and the eligibility criteria for inclusion were detailed in the Operational Definition. The general data acquisition process is presented in Supplementary file Figure 1.

### Data analysis

*Spatial distribution analysis*

The Global Moran’s Index is a commonly used statistic to measure spatial autocorrelation. It describes the average degree of correlation of all spatial units with their neighbors on the whole region by calculating the similarity of spatial unit attribute values with neighboring units. The Global Moran’s Index ranges from -1 to +1, where values above zero indicate positive spatial correlation, zero suggests no correlation, and below zero indicates negative correlation. Using ArcMap10.8 software to perform the calculations, we revealed the spatial aggregation of provincial tobacco sponsorship expenditures from 2015 to 2022. We constructed the spatial weights matrix using first-order Queen contiguity (Provincial administrative units sharing a border or a single vertex are neighbors). Hainan, having no land neighbors, was automatically identified as an island and its weights were set to 0 after row-standardization.

*Topic modeling*

Latent Dirichlet Allocation (LDA) topic modeling was used to capture the thematic focus of tobacco sponsorship website publicity.

To ensure semantic integrity, topic modeling was performed entirely on the original Chinese corpus. The preprocessing pipeline involved: 1) Text Cleaning – removing non-textual characters, HTML tags, and URLs; 2) Segmentation – utilizing the Jieba library for precise Chinese word segmentation; and 3) Filtering – removing stop words using a Chinese stop-word list (Supplementary file) to minimize noise.

The weight and clustering of high-frequency words in tobacco-sponsored texts was calculated by term frequency–inverse document frequency (TF-IDF). To determine the optimal number of topics, we calculated both perplexity and coherence scores for candidate models ranging from 1 to 15. 4 as the optimal solution, as it ensured the most semantic interpretability. Subsequently, we analyzed the temporal changes in topic popularity.

The entire analysis was implemented in Python, with visualizations generated using Origin. For reporting purposes, the resulting topic labels were translated into English.

*Textual coding*

Cigarette production (CP)

Restricted in traditional advertising, tobacco companies often resort to sponsorship for brand presence. Cigarette production (CP) has long been proved as an indispensable contextual variable for smoking through two channels. From a consumer market perspective, increased production heightens product exposure and reduces prices, enhancing accessibility^17^. From the regulatory perspective, the state-owned cigarette manufacturing activities are ‘key generator of local government revenue’, undermining the incentives for regulations change with industrial economic reliance^17^. Given the close link between production and consumption, cigarette production was used as a proxy for sales, as validated by prior literature^24^.

Smoke-free law efficacy (SLE)

Smoke-free laws may reduce public smoking and hinder smoker attraction. Research from Spain has shown that more stringent legislation contributes to a ‘homogenization of cigarette sales’, limiting brand differentiation strategies^25^. This finding implies that smoke-free law efficacy, defined by their coverage and duration, may also shape the sponsorship.

Government-industry connection (GIC)

Political activity has long been a critical mechanism through which tobacco companies safeguard their commercial interests. By cultivating a favorable policy environment, they are able to obstruct or delay tobacco control efforts^26^. Considering such backdrop, sponsorship disguised as philanthropy, facilitates agenda setting in tobacco control policies and expands access to policymakers, for example, prominent political figures may be invited to present and recommend such events^16^. Consequently, these connections serve as an informal yet potent indicator of regulatory strength.

Production chain linkage (PCL)

Supply chain management remains pivotal to tobacco farming decisions. Usually, companies facilitate production by providing inputs like fertilizers and pesticides, alongside incentives such as loans, price guarantees, and medical insurance^27^. These provisions nurture loyalty and a perceived ‘family’ bond, securing long-term commitment. Therefore, sponsorship targeting supply chain stakeholders may be understood as a mechanism for reinforcing the production chain linkage, ensuring continuity in tobacco supply and stabilizing the upstream production network through workforce morale enhancement. Supplementary file Table 1 presents the abbreviations referenced in this study, their standard names, and the corresponding measurement indicators.

GIC and PCL were operationalized through coding of corporate website texts. After discussion, three authors reached a consensus on the definition of sponsorship in website publicity and the standards of marking variables. Randomly selecting 10% samples for pre-coding, three coders achieved an inter-coder reliability of 0.79 assessed by Fleiss’ kappa, ensuring good consistency and accuracy throughout the coding process. Subsequently, coders collaboratively coded all texts.

### Statistical modeling

Based on the coding results, we analyzed the effects of CP, SLE, GIC, and PCL on sponsorship amounts (SA) using STATA 17.0. Given that this study aims to estimate the marginal changes in tobacco sponsorship amounts of provincial administrative units and their linear associations with legislation in force and cigarette production, ordinary least squares (OLS) regression was employed as it provides unbiased, easily interpretable coefficients with minimum variance. To mitigate confounding bias inherent in observational data, we identified GIC and PCL as potential confounders. These variables likely influence both the primary predictors (SLE and CP) and the dependent variable (SA). Consequently, we constructed three sequential models: Model 1 estimates the total effect of the core exposure variables without adjustment. Model 2 incorporates GIC and PCL as control variables to isolate the ‘net effects’ of SLE and CP. Model 3 introduces ‘tobacco farmer mentions’ as a mediator on this confounder-adjusted baseline to ensure the indirect effect estimation conforms to causal inference protocols. Furthermore, to control for potential temporal effects, both models included year-fixed effects; model fit was assessed using R^2^ (coefficient of determination), which indicates the proportion of provincial-level variation in tobacco sponsorship expenditure explained by the included predictors.

Second, we examined the underlying influence mechanism. We first assessed the direct effects of SLE, CP, GIC, and PCL on SA, based on the finding of which we evaluated the effects of tobacco farmers as the mediating variable. All statistical tests were conducted using a two-tailed significance threshold of p<0.05.

Finally, to ensure the reliability of our baseline findings, we implemented three sequential robustness checks: first, we re-estimated the models using heteroscedasticity-robust standard errors to prevent potential bias in significance levels; second, standard errors were clustered at the provincial level to account for potential within-province autocorrelation over time; and third, we performed a lagged effect analysis by replacing ‘Smoke-free Law Efficacy’ with its one-period lagged version to test the persistence of policy impacts and mitigate potential endogeneity. These supplementary tests were conducted to confirm that the core conclusions of the aforementioned models remain robust across different specifications.

## RESULTS

### Sponsorship amount

*Descriptive analysis*

Sponsorship amounts nationwide from 2015 to 2022 are shown in Supplementary file Figure 2. It plummeted sharply in 2017, then slowly rebounded, and fell again in 2021.

Table 1 illustrates the distribution of sponsorship amounts of provincial administrative units in China, revealing notable regional disparities. Overall, with reference to the geographical location of each province in China, the southwest region has the highest sponsorship amount, with Yunnan Province ranking first in the country. The central region, represented by the closely linked two provinces, Hunan and Jiangxi, followed not far behind. Northeast China, consisting of such as Heilongjiang, Jilin, and Liaoning, is significantly lower than other regions.

**Table 1:** Ranking and inter-annual variability of tobacco sponsorship amount across 31 Chinese provincial administrative units, based on cumulative percentage, 2015–2022 (N=248)

*Provincial administrative units*	*Percentage*	*Ranking*	*SD*
Yunnan	24.10	1	49088.57
Hunan	12.11	2	12324.39
Zhejiang	8.25	3	19167.87
Jiangxi	7.49	4	8242.15
Fujian	5.97	5	7979.69
Chongqing	5.10	6	13963.39
Guizhou	4.91	7	4292.89
Hubei	4.73	8	5032.97
Anhui	2.80	9	13148.70
Shanghai	2.62	10	2006.42
Shaanxi	2.49	11	4084.22
Xinjiang	2.42	12	4867.23
Guangdong	2.20	13	1683.74
Xizang	2.15	14	13097.12
Sichuan	1.70	15	1412.68
Hebei	1.54	16	5727.85
Guangxi	1.31	17	2483.47
Shandong	1.23	18	2224.96
Henan	1.19	19	2305.07
Jiangsu	1.14	20	488.72
Shanxi	1.04	21	2842.19
Gansu	0.93	22	1038.63
Hainan	0.75	23	3673.51
Inner Mongolia	0.60	24	1513.75
Liaoning	0.37	25	257.39
Jilin	0.29	26	619.19
Heilongjiang	0.23	27	324.28
Beijing	0.12	28	234.58
Qinghai	0.12	29	263.24
Ningxia	0.10	30	133.74
Tianjin	0.03	31	123.32
Taiwan	-	-	-
Hong Kong	-	-	-
Macau	-	-	-

Rankings are based on cumulative provincial amounts during 2015–2022. Consistent with China Tobacco Yearbooks, we included statistics for 31 provincial administrative units. SD: standard deviation of annual provincial sponsorship over 8 years; larger values indicate greater year-to-year variability.

*Moran’s Index analysis*

The results of the Global Moran’s Index are shown in Table 2. From 2016 to 2020 and in 2022, the consistently positive Moran’s I values (greater than 0.1, p<0.05, Z≥2.17) indicate a strong spatial association between SA and geographical location, reflecting significant regional agglomeration.

**Table 2:** Global Moran’s I of China tobacco sponsorship amount across 31 Chinese provincial administrative units, 2015–2022 (N=248)

*Year*	*Moran’s I*	*Z*	*p*
2015	0.032	1.451	0.147
2016	0.142	2.331	**0.020**
2017	0.269	4.052	**0.000**
2018	0.238	3.891	**0.000**
2019	0.106	2.189	**0.029**
2020	0.112	2.499	**0.012**
2021	0.005	0.588	0.556
2022	0.096	2.170	**0.030**

Moran’s Index is a spatial statistic that measures the degree of spatial autocorrelation, indicating whether similar values cluster or disperse across a geographical area. Z>1.96 indicates significant spatial autocorrelation; Two-tailed p<0.05 is used as the significance threshold.

The non-significant Moran’s I in 2021 (I=0.005, p=0.556) coincides with the cancellation of most on-site sponsorship events during the second year of the COVID-19 pandemic. A leave-one-out diagnostic shows that the drop in national spatial autocorrelation was driven mainly by the three largest tobacco-producing provinces – Yunnan, Hunan and Guizhou – whose 2021 sponsorship expenditures all fell sharply compared with 2020, eroding the previously stable high–high cluster in Southwestern China.

*Topic modeling*

Table 3 presents the topics generated by LDA (the original Chinese version of the table is given in Supplementary file Table 2). We identified two key clusters as focal points in Tobacco Sponsorship Publicity: 1) Social Responsibility and Public Affairs Involvement; and 2) Industry and Business Development.

**Table 3:** Keywords, thematic labels, and topic intensities on LDA topic modeling analysis of tobacco sponsorship publicity articles collected from four major Chinese tobacco industry websites, 2015–2022 (N=2429)

*Topic*	*Keywords*	*Topic label*	*Intensity*
1	0.018 × ‘Pandemic’ + 0.016 × ‘Prevention and Control’ + 0.008 × ‘Donation’ + 0.007 × ‘Pandemic prevention’ + 0.006 × ‘Nucleic Acid’ + 0.005 × ‘Supplies’ + 0.005 × ‘Love’ + 0.005 × ‘Volunteer’ + 0.005 × ‘Test’ + 0.005 × ‘Activity’ + 0.004 × ‘Anti-Pandemic’ + 0.004 × ‘Mask’ + 0.004 × ‘Pneumonia’ + 0.004 × ‘Doing well’ + 0.004 × ‘Society’ + 0.004 × ‘Donate’ + 0.004 × ‘Employers’ + 0.004 × ‘Sympathy’ + 0.003 × ‘Cadres’ + 0.003 × ‘Practical actions’	Natural and Health Disaster Response	0.1737
2	0.012 × ‘Tobacco leaf’ + 0.009 × ‘Tobacco farmers’ + 0.009 × ‘Industry’ + 0.007 × ‘Poverty elimination’ + 0.006 × ‘Plant’ + 0.006 × ‘Poverty Alleviation’ + 0.006 × ‘Development’ + 0.005 × ‘Revitalization’ + 0.005 × ‘Produce’ + 0.004 × ‘Assistance’ + 0.004 × ‘Toasted tobacco’ + 0.004 × ‘Fight critical battles’ + 0.004 × ‘Construction’ + 0.004 × ‘Cooperatives’ + 0.003 × ‘Income generation’ + 0.003 × ‘Agriculture’ + ‘0.003 × ‘Poverty-stricken household’ + 0.003 × ‘Reservoir’ + 0.003 × ‘Land’ + 0.003 × ‘Technology’	Tobacco Product Manufacturing and Influence	0.2793
3	0.024 × ‘Poverty alleviation’ + 0.019 × ‘Poverty elimination’ + 0.013 × ‘Assistance’ + 0.013 × ‘Poverty-stricken household’ + 0.012 × ‘Fight critical battle’ + ‘0.009 × ‘Sympathy’ + 0.008 × ‘Visit’ + 0.006 × ‘Detailed’ + 0.006 × ‘Fixed-point’ + 0.006 × ‘Blood donation’ + 0.005 × ‘Donation’ + 0.005 × ‘Situation’ + 0.005 × ‘Relief supplies’ + 0.005 × ‘Targeted’ + 0.005 × ‘Activity’ + 0.004 × ‘Battle against poverty’ + 0.004 × ‘Win’ + 0.004 × ‘Team’ + 0.004 × ‘Achievement’ + 0.004 × ‘People in need’	Poverty Alleviation and Policy Response	0.0937
4	0.006 × ‘Client’ + 0.005 × ‘Retail’ + 0.004 × ‘Manage’ + 0.003 × ‘Terminals’ + 0.003 × ‘Stores’ + 0.003 × ‘Agricultural network’ + 0.003 × ‘Services’ + 0.002 × ‘Children’ + 0.002 × ‘Merchandise’ + 0.002 × ‘Brand’ + 0.002 × ‘Activity’ + ‘0.002 × ‘Customer’ + 0.002 × ‘Crowd’ + 0.002 × ‘Learn’ + 0.002 × ‘Spend’ + 0.002 × ‘Account Manager’ + 0.002 × ‘Red’ + 0.002 × ‘Marketing’ + 0.002 × ‘Livestreaming’ + 0.002 × ‘Build’	Customer Service and Brand Building	0.4525

The numbers preceding the keywords represent the weights associated with each term. A higher value indicates a stronger contribution of the keyword to the definition of the corresponding topic. Topic popularity: sum of keyword probabilities.

The first cluster includes Natural and Health Disaster Response (Topic 1) and Poverty Alleviation and Policy Implementation (Topic 3). This cluster primarily discusses how tobacco sponsorship activities can be used through quick responses to current priority public concerns. In routine operations, the tobacco industry emphasizes its participation in the strategic priorities of the national development agenda, particularly in poverty alleviation. While in response to unexpected health crises or natural disasters, tobacco companies proactively position themselves as contributors to relief efforts.

The second cluster includes Tobacco Product Manufacturing and Influence (Topic 2) and Customer Service and Brand Building (Topic 4). This cluster highlights how tobacco companies allocate sponsorship funds across the entire supply chain. The production and manufacturing of tobacco products reflect a focus on core business operations, particularly, tobacco farmers and agricultural practices. Moreover, tobacco companies further enhance their influence by engaging in customer service and marketing activities, expanding and upgrading distribution channels.

**Figure 1:**
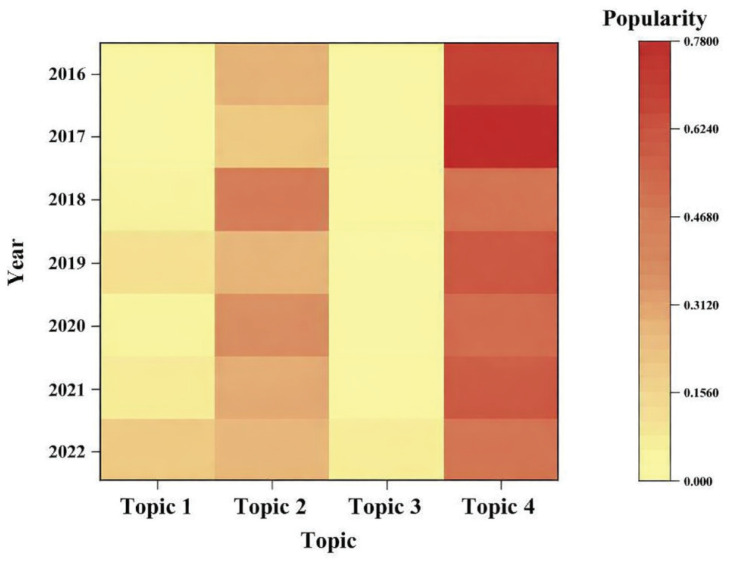
Temporal trends in topic popularity of four primary topics derived from LDA analysis for tobacco sponsorship publicity of relevant Chinese articles, 2015–2022 (N=2429). Topic 1: Natural and Health Disaster Response; Topic 2: Tobacco Product Manufacturing and Influence; Topic 3: Poverty Alleviation and Policy Implementation; Topic 4: Customer Service and Brand Building

Figure 1 illustrates temporal variations in topic popularity, with darker colors and higher values signifying greater importance of specific topics.

Generally, the cluster of Industry and Business Development has consistently been a focal point of tobacco industry sponsorship. Following the introduction of the Charity Law in 2016, Topic 4, despite a subsequent recovery, experienced a significant 2-year decline. In response, the industry redirected its focus toward investing in the internal growth and flexibility to circumvent regulatory constraints, as demonstrated by the simultaneous rise in Topic 2.

The Social Responsibility and Public Affairs Involvement cluster is closely linked to key policy developments and the broader societal context. Topic 3 maintained upward momentum through 2022 (except 2019), in tandem with a series of national official documents supporting poverty alleviation, showing tobacco companies’ political resonance. Moreover, the COVID-19 pandemic catalyzed a sharp surge in Topic 1.

*Factors affecting tobacco sponsorship*

Correlation analysis and covariance test reveal a strong positive correlation between SA and both CP and GIC is identified, while CP also exhibits a strong positive correlation with GIC and PCL. Additionally, GIC is positively correlated with PCL. The variance inflation factor (VIF) values all <10 indicated that there is no multicollinearity and that the model is well constructed.

To further examine the impact of each variable on sponsorship amounts, we performed an ordinary least squares (OLS) regression analysis (Table 4). The results in column (1) indicate that SLE has high negative impacts on SA, while CP has some degree of positive effect on SA. Column (2) includes GIC and PCL as control variables. The results indicate that SLE and CP remain significant, but neither GIC nor PCL significantly affects SA.

**Table 4:** Ordinary least squares regression results examining factors associated with provincial tobacco sponsorship amounts in China, 2015–2022 (N=232)

	*Model 1*		*Model 2*		*Model 3*	
	*Sponsorship amounts (no control variables added)*	*95% CI*	*Sponsorship amounts (control variables added)*	*95% CI*	*Mentioning tobacco farmers*	*95% CI*
Smoke-free law efficacy	-0.149*** (-3.287)	-0.238 – -0.060	-0.149*** (-3.216)	-0.240 – -0.058	-0.351*** (-2.747)	-0.603 – -0.099
Cigarette production	0.001*** (13.321)	0.001–0.002	0.001*** (12.990)	0.001–0.002	0.001** (2.206)	0.000–0.001
Government-industry connection			-0.002 (-0.237)	-0.022–0.017	0.138*** (5.024)	0.084–0.192
Production chain linkage			0.000 (0.016)	-0.030–0.030	0.338*** (8.090)	0.255–0.420
Constant	0.018 (0.107)	-0.312–0.347	0.017 (0.103)	-0.314–0.349	0.209 (0.449)	-0.708–1.126
Time effect	year		year		year	
Individual effect	year		year		year	
R^2^	0.442		0.443		0.832	
F	90.672		45.077		281.935	

Model 1: crude effects. Model 2: adds the confounders of government-industry connection and production chain linkage. Model 3: tests mediation of ‘tobacco-farmer mentions’. Coefficients are ordinary least squares regression estimates; t-values in parentheses. Model 1 contains only core independent variables and excludes government-industry connection and production chain linkage; consequently, the corresponding cells in the first column are left blank to maintain the integrity of the table. Due to missing data in certain control variables, the regression analysis employed the full case analysis method, yielding an effective sample size of 232 observations.

*** p<0.01,

** p<0.05,

* p<0.10.

We further explored the mechanisms that influence SA through the mediating effect two-step approach. As SLE (β= -0.149; 95% CI: -0.238 – -0.060, p<0.01) and CP (β= 0.001; 95% CI: 0.001–0.002, p<0.01) are calculated to have negative and positive significant effects on SA, we delineated the particular mention of tobacco farmers from the PCL variable as a mediating factor, based on our preliminary observation from coding that tobacco farmers frequently co-occur with government and tobacco companies in sponsorship texts. Column (3) confirms that the number of mentioning tobacco farmers has a significant negative effect on SLE (indirect effect= -0.351; 95% CI: -0.603 – -0.099, p<0.01) and a positive effect on CP (indirect effect=0.001, 95% CI: 0.000–0.001, p<0.05), GIC (indirect effect=0.138; 95% CI: 0.084–0.192, p<0.01), and PCL (indirect effect= 0.338; 95% CI: 0.255–0.420, p<0.01). Therefore, SLE and CP not only affect SA directly, but also indirectly through the mediation of tobacco farmers.

*Robustness checks*

To verify the reliability of the baseline regression findings, this study conducted three sets of robustness tests. First, we re-estimated the models using heteroscedasticity-robust standard errors to mitigate potential bias in significance levels. Second, standard errors were clustered at the provincial level to account for unobserved heterogeneity and potential autocorrelation within provincial administrative units. Finally, the independent variable SLE was replaced with its one-period lagged value to examine the temporal persistence of policy impacts and address potential endogeneity.

As presented in Supplementary file Table 3, the results across all three specifications remained highly consistent with the baseline findings: CP maintained a significantly positive impact on sponsorship expenditure (p<0.01), while the SLE continued to exhibit a significant negative effect (p<0.05). Furthermore, GIC and PCL remained statistically non-significant in all models. Collectively, these results demonstrate that the primary conclusions of this study are robust under various error structures and dynamic specifications.

## DISCUSSION

### Tobacco sponsorship is profit-seeking in essence

Empirical findings reveal the commercial logic underpinning tobacco industry sponsorship activities. Regression analysis shows that regional cigarette production is significantly and positively associated with tobacco sponsorship amounts. Topic modeling and content analysis further locate the discursive emphasis on production chain stakeholders. With business priorities prevailing, the results together suggest that such sponsorship is more of calculated investments in market expansion and supply chain consolidation, rather than mere disinterested philanthropy for traditionally assumed beneficiaries like vulnerable populations and economically disadvantaged students^24^.

Among the complicated market chain, tobacco farmers and retailers are identified as major beneficiaries. Sponsorship activities are highly concentrated in tobacco-growing regions due to their help to secure farmer loyalty and stabilize supply chains^14^. Furthermore, these initiatives encourage cropping strategies combining tobacco with other agricultural products, ostensibly promoting crop diversification, but primarily functioning as risk mitigation mechanisms. Since tobacco is hindered by its seasonal growth cycle, tobacco farming is often a part-time or temporary occupation rather than stable employment^27^. When economic considerations remain the primary determinant of cultivation decisions, planting supplementary crops may effectively reduce agricultural income volatility and ensure continued land allocation to tobacco cultivation despite fluctuating productions.

Retailers, as the direct touch-points between tobacco companies and consumers, play an important role in shaping consumer experience, which directly impacts cigarette sales. Therefore, on the one hand, companies are investing in the expansion of their retail outlets, whilst on the other, they aim to enhance customer engagement by providing targeted training programmes^28^. Positioning the increase of profits as the central objective, these activities all serve to stabilize and expand production and selling networks essential for profit maximization.

### Diverse strategies of action and publicity adopted to cover the commercial nature

Financial support is the most common sponsorship strategy. However, with the gradual implementation of the TAPS ban, sponsorship activities have become increasingly indirect and less identifiable. In China, leveraging their state ownership, tobacco companies align their sponsorship activities and publicity with national policy priorities and public concerns to cover the commercial need.

The increases in Topic 1 (Natural and Health Disaster Response) and Topic 3 (Poverty Alleviation and Policy Implementation) fully reflect the industry’s adaptability. In routine operations, tobacco companies have linked their sponsorship efforts to the national strategic focus on rural revitalization and poverty alleviation to enhance their political access. Through infrastructure construction, the purchase of poverty alleviation products, and the donation of living supplies, these sponsorship activities help to improve the income and living conditions of impoverished populations, serving as subsidies to help alleviate local fiscal and developmental gaps. Moreover, tobacco companies have also shown a remarkable ability to adapt their promotional focus in response to current events. For instance, following the COVID-19 outbreak, tobacco companies quickly started their anti-pandemic sponsorship to donate emergency supplies and medical equipment. Framed with philanthropic terminology such as ‘aid’ and ‘donation’ in their publicity, the aforementioned practices reflect global trends in the tobacco industry’s response to regulatory constraints and heightened public scrutiny, demonstrating the industry’s maneuver in using socially relevant topics to build public tolerance by beautifying its image.

Moreover, due to their blurred institutional boundaries with government agencies, tobacco companies may face fewer restrictions and more easily cooperate with state-led programs. Such targeted sponsorship allows companies to build broad connections with different apparatus of government, and construct discourse alliances that conflate tobacco with a broader vision of national development and prosperity goals^29^. By doing so, they frame themselves as supporters instead of opponents of national development, obscuring the fact that they take advantage of these initiatives to increase the cultivated area and the number of tobacco farmers^30^.

Though often self-claimed as CSR efforts, sponsorship targeting tobacco farmers and retailers, key components of the industrial chain, is essentially a strategic business investment. Now, China had already amassed a substantial number of tobacco farmers and retailers, comprising a significant share of the industry’s workforce^31^. Topic modeling reveals that this group is closely associated with socially disadvantaged populations, such as individuals with disabilities and the impoverished. By doing so, tobacco companies have thus maneuvered the expansion of production and sales as employment promotion and poverty alleviation. Furthermore, we identify a positive relationship between cigarette production and sponsorship amounts, indicating that tobacco companies adopt a similar behavioral logic to divert attention from the health risks associated with tobacco use.

### Targeted national legal policies are necessary

Article 13 of the WHO FCTC requires a comprehensive ban on sponsorship^1^. As a Party to the FCTC, China has already scored 9/10 in the annual evaluation of compliance with indirect bans and introduced the Charity Law in 2016, which bans tobacco sponsorship^10^. Following the introduction, both sponsorship amounts and publicity centering on brand promotion experienced a decline in 2017 and 2018. Regression analyses further indicate that legislation banning smoking in public places is associated with a significant reduction in sponsorship amounts, reinforcing the role of legislation as an effective tool for controlling tobacco sponsorship. Although these statistical associations exist, it is important to note that the analysis reflects the existence of legislation rather than its actual enforcement. Therefore, we admit that the result reflects more of an association with the legal framework’s presence rather than a direct measure of implementation effectiveness.

However, the results still indicate that regulatory loopholes persist as this decline was short-lived, with sponsorship levels rebounding rapidly in 2018 and continuing until the COVID-19 pandemic negatively impacted tobacco production and the economy in 2020. In terms of specificity, it is essential to explicitly define and penalize all sponsorship activities by tobacco companies. A major criticism of the current Charity Law is that its lack of a clear definition of tobacco sponsorship and failure to specify penalties for violations allow tobacco companies to exploit CSR as a disguise. And these loopholes persist, despite the revision of the Charity law in 2023. Evidence from local authorities in England suggests that standardized guidance from the national government, exemplified by clear definitions and application, may help reduce ambiguity and enable more consistent and targeted action^32^. Moreover, experiences from Spain demonstrate that stricter TAPS bans are more effective in regulating the market behavior of tobacco companies. Partial bans were shown to be insufficient, while comprehensive prohibitions led to measurable impacts on reducing cigarette sales^33^. Fines have also been shown to be effective in reducing smoking prevalence^34^. Similarly belonging to the TAPS ban, a more explicit, strict and comprehensive tobacco sponsorship law – one that clearly defines tobacco sponsorship and strengthens legal penalties for violations – may also work for combating tobacco sponsorship in China.

In terms of scope, the introduction of nationwide legislation is essential as a top-level guidance and guarantee. Provincial sponsorship amounts have a high spatial concentration in the vast majority of years, which is shown by the spatial auto-correlation analysis results, and pilot anti-tobacco efforts primarily limited to the local level together suggest the necessity of implementing tobacco control legislation on a larger geographical scale, ideally nationwide. Previous research has also shown that national bans, which have a wider coverage of all locations, tend to achieve larger effect sizes than local ones^35^. Such legislation would not only influence the government’s official stance on tobacco control but also ensure more consistent enforcement across different administrative levels.

In the legislative process, the tobacco industry has often obstructed, delayed, and weakened national tobacco control legislation^36^. To create a more conducive environment for the enactment of such legislation, a series of complementary measures are necessary. Efforts to secure technical and financial support from professional public health and tobacco control organizations, foster effective civil society cooperation, and build public opinion support may all help to advance comprehensive tobacco sponsorship bans. For example, the media in Bangladesh have played an active role in monitoring violations and enforcement, engaging policymakers for multi-sectoral cooperation, and raising public awareness of the issue^18^.

### Limitations

The study has several limitations. First, its reliance on tobacco industry-reported sponsorship data from the China Tobacco Yearbooks may introduce reporting bias as self-reported industry data from companies limit complete objectivity. Government-industry connection and production chain linkage were derived from corporate publicity texts and should be viewed as surrogate markers rather than direct behavioral measures; selective disclosure or framing bias could attenuate their validity. Future studies may cross-verify data for better objectivity. Second, as an observational study, it identifies associations but can hardly establish causality between legislation, production, and sponsorship. The direction of influence between sponsorship amounts and anti-tobacco legislation may be reversed, such that provinces with higher sponsorship could be less likely to enact stringent laws. Furthermore, residual confounding, for example from shifts in corporate strategy or regional economic policies, may also influence the observed relationships. While longitudinal data and regression mitigate this issue, causal influence requires quasi-experimental designs. Third, our measure of smoke-free policies counted enacted laws but did not capture their variations in enforcement intensity and levels, potentially biasing estimates of policy effectiveness. Finally, the generalizability of our findings warrants consideration. China operates under a unique state-owned tobacco monopoly system, where the specific forms and motivations of sponsorship observed in this context may differ from those in countries with privatized tobacco markets. However, the sponsorship activities and their publicity patterns revealed in this study may still offer insights for global tobacco control, particularly for other nations grappling with indirect sponsorship activities. Future research could explore how these findings might extend to other regions, and conduct comparisons in contexts with varying levels of regulatory enforcement to improve the worldwide implementation of the FCTC.

## CONCLUSIONS

China’s tobacco companies have strategically framed their sponsorship behavior and publicity in ways that resonate with broader narratives of national development. By doing so, they have not only improved their social image, but also secretly accumulated resources for their business development, while simultaneously increasing the monetary reliance of both the public and government on them. Although these efforts indeed contribute to social welfare and community development, they are in essence aiming at evading regulations to enhance profits and reputation. Therefore, it is necessary to remain aware of this reality and, in accordance with the FCTC, implement a comprehensive nationwide ban on tobacco sponsorship. However, to effectively support and refine such policy interventions, future research should prioritize more comprehensive data and methodologies capable of establishing causality, such as experimental or quasi-experimental designs, to more robustly assess the impacts of tobacco sponsorship bans. Additionally, exploring the broader socio-economic and political contexts of tobacco control will be critical in informing effective and context-specific policies. By addressing these research gaps, we can accumulate the evidence needed to strengthen legislative frameworks and ensure the implementation of impactful tobacco control measures consistent with the WHO FCTC.

## Supplementary Material



## Data Availability

Data sharing is not applicable to this article as no new data was created.
