# Supplementary material for: Improving legislation is a necessity: A multi-method quantitative analysis of tobacco sponsorship and publicity in China
Source: Tob Induc Dis. 2026 Mar 24;24:10.18332/tid/217354. doi: 10.18332/tid/217354 (PMC13012016; doi:10.18332/tid/217354)
Supplement: Supplementary file 1 [file TID-24-44-s1.pdf]

Supplementary materials

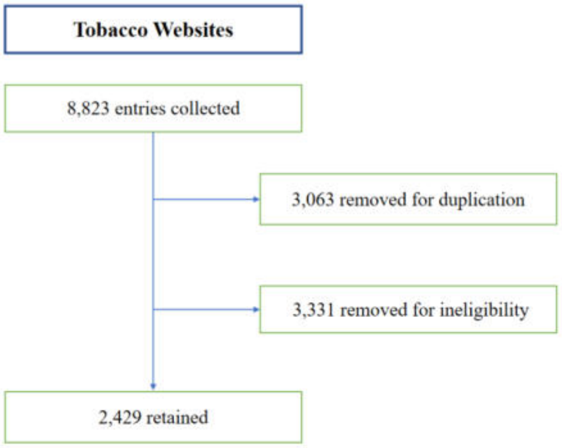

Supplementary Figure 1 Flowchart of the selection of articles on the database of tobacco sponsorship in China, 2015–2022 (N = 2,429)

Supplementary Table 1 List of Abbreviations and Their Meanings

**Category**	**Variable**	**Abbreviation**	**Operationalization**
Dependent	Sponsorship amounts	SA	Annual sponsorship amount of each provincial administrative units*
Independent	Cigarette production	CP	Annual cigarette production of each provincial administrative units (in billions of cigarettes)
Independent	Smoke-free law efficacy	SLE	The number of provincial/municipal smoke-free laws and regulations in force
Independent	Government-industry connection	GIC	Frequency of government entity mentions in publicity texts (If several different government agencies appear in a single text, each instance shall be counted separately. References to the same government body within a single report shall be counted as a single occurrence)
Independent/Intermediary	Production chain linkage	PCL	Frequency of tobacco workforce (including tobacco farmers, tobacco retailers, and tobacco company staff) mentions in publicity texts（Where multiple distinct categories of tobacco workforce are mentioned within a single report, each category shall be counted separately. Repeated mentions of the same category within a single report shall be counted as a single occurrence)

*Note: SA in millions of Renminbi (CNY, Chinese Yuan)

1

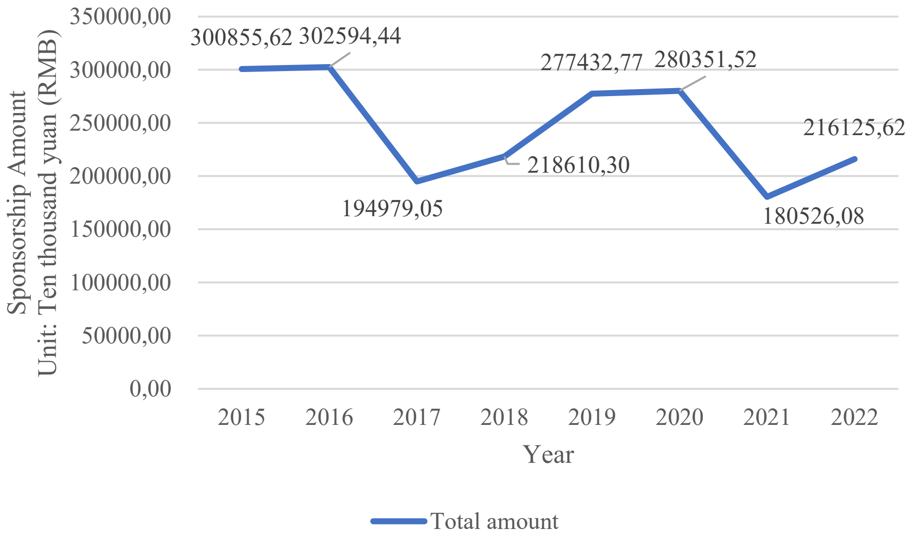

Supplementary Figure 2 Tobacco sponsorship amount in China from 2015 to 2022, based on industry reports aggregated across 31 provincial administrative units (N=248)

Supplementary Table 2 Original Chinese keywords, thematic labels, and topic intensities on LDA topic modeling analysis of tobacco sponsorship publicity articles collected from four major Chinese industry websites (2015–2022, N=2429)

Topic	Keywords	Topic Label	Intensity
1	'0.018*"疫情" + 0.016*"防控" + 0.008*"捐款" + 0.007*"防疫" + 0.006*"核酸" + 0.005*"物资" ' '+ 0.005*"爱心" + 0.005*"志愿者" + 0.005*"检测" + 0.005*"活动" + 0.004*"抗疫" + ' '0.004*"口罩" + 0.004*"肺炎" + 0.004*"做好" + 0.004*"社会" + 0.004*"捐赠" + 0.004*"职工" ' '+ 0.004*"慰问" + 0.003*"干部职工" + 0.003*"实际行动"	Natural and Health Disaster Response	0.1737
2	'0.012*"烟叶" + 0.009*"烟农" + 0.009*"产业" + 0.007*"脱贫" + 0.006*"种植" + 0.006*"扶贫" ' '+ 0.006*"发展" + 0.005*"振兴" + 0.005*"生产" + 0.004*"帮扶" + 0.004*"烤烟" + ' '0.004*"攻坚" + 0.004*"建设" + 0.004*"合作社" + 0.003*"增收" + 0.003*"农业" + ' '0.003*"贫困户" + 0.003*"水库" + 0.003*"土地" + 0.003*"技术"	Tobacco Product Manufacturing and Influence	0.2793
3	'0.024*"扶贫" + 0.019*"脱贫" + 0.013*"帮扶" + 0.013*"贫困户" + 0.012*"攻坚" + ' '0.009*"慰问" + 0.008*"走访 " + 0.006*"详细" + 0.006*"定点" + 0.006*"献血" + 0.005*"捐赠" ' '+ 0.005*"情况" + 0.005*"慰问品" + 0.005*"精准" + 0.005*"活动" + 0.004*"攻坚战" + ' '0.004*"打赢" + 0.004*"工作队" + 0.004*"成果" + 0.004*"困难群众"	Poverty Alleviation and Policy Implementation	0.0937
4	'0.006*"客户" + 0.005*"零售" + 0.004*"经营" + 0.003*"终端" + 0.003*"店铺" + 0.003*"农网" ' '+ 0.003*"服务" +	Customer Service and Brand Building	0.4525

	0.002*"孩子" + 0.002*"商品" + 0.002*"品牌" + 0.002*"活动" + ' '0.002*"顾客" + 0.002*"群众" + 0.002*"学习" + 0.002*"消费" + 0.002*"客户经理" + ' '0.002*"红色" + 0.002*"营销" + 0.002*"直播" + 0.002*"建设"		

Supplementary Table 3 Robustness checks for the main regression model using alternative standard error estimations and a lagged variable specification (2015–2022; N=232 for columns 1-2, N=203 for column 3).

	(1)<br>Robust Standard Errors	(2)<br>Clustered Clustered Standard Errors	(3)<br>Lagged Effect
Smoke-free law efficacy	-0.149**	-0.149***	
	(-2.508)	(-3.216)	
Cigarette production	0.001***	0.001***	0.001***
	(4.116)	(12.990)	(4.585)
Government-industry connection	-0.002	-0.002	-0.000
	(-0.271)	(-0.237)	(-0.006)
Production chain linkage	0.000	0.000	0.001
	(0.022)	(0.016)	(0.062)
Lagged Smoke-free law efficacy			-0.130**
			(-2.564)
Constant	0.017	0.017	0.145
	(0.123)	(0.103)	(1.200)
Year Fixed Effects	year	year	year
Province Fixed Effects	year	year	year
N	232	232	203
R^2	0.443	0.443	0.421
F	8.625	45.077	8.015

Note: Smoke-free law efficacy is the impact of the number of prefecture-level cities enacting laws and regulations in the previous period on tobacco sponsorship amounts in the current period. This table indicates that for each additional unit increase in the number of prefecture-level cities enacting laws and regulations in the previous period, the average tobacco sponsorship amount in the current period decreases by approximately 0.130 units.

Lagged Smoke-free law efficacy is the one-period lag of Smoke-free law efficacy, capturing the delayed impact of smoke-free law efficacy on tobacco sponsorship amounts.

Robust Standard Errors, providing more reliable standard error estimates to assess variable significance.

Clustered Standard Errors, account for correlated errors within pre-defined groups (e.g., individuals, regions), yielding more accurate significance tests when observations within clusters are not independent.

Lagged Effect refers to the delayed impact of an explanatory variable on the dependent variable, captured by including the variable's prior-period values to account for temporal delays in its influence.

t-values in parentheses; ***p<0.01, **p<0.05, *p<0.10

© 2026 Sun S. et al.
